# Letter from Dr. Peebles on Articulating Sets of Teeth

**Published:** 1849-04

**Authors:** H. E. Peebles


### LETTER FROM DR. PEEBLES ON ARTICULATING SETS OF TEETH.

BY H. E. PEEBLES.

After trying various plans, of others and my own, to facilitate the labor of making an antagonising cast, and finding objections to all, I finally fell upon the following, tried it, and adopted it.

Two white pine blocks, one and a half inches square and three inches long, one rabbeted one-half inch by one-thirtieth of an inch, and held together with a good brass butt hinge in such manner that when folded upon each other the rabbet is in the centre; a piece of thick tin-plate three by ten inches, with two small holes near each end, and two others near the centre; another piece of tin one inch by three inches. After striking up the plates, and obtaining the impressions as usual, bend the large tin-plate in form of the letter U, place the wax and plates in the centre of the tin with the labial side of the wax next the tin, and confine it there by thrusting two pins through the two central holes in the tin into it (the wax); then apply the blocks, with the small tin-plate held between them, in the rabbet, with half its width projecting toward the wax, and push them gently up till the plate touches the wax with the impressions, and confine the blocks by driving a small tack in each end, through the holes in the large tin plate, (it is well to arm these blocks with a few tacks driven half their length into the sides next the wax, to hold the plaster firm to them.) Thus then you have all ready to pour in your plaster on one side, and if it is of proper consistence, you may turn it over and fill the other side in five minutes after. Thus you save much time and trouble; and after standing twenty-five minutes more, you may draw the tacks and pins that hold the blocks and wax, take off the tin-plate, place the whole mass in warm water, and in a few minutes separate your casts, take out your wax, and you have a beautiful, accurate, and convenient antagonising cast. The same tin-plates answer, and so with the blocks, if you choose to separate them from the plaster for any number of jobs; but I prefer using the same blocks only once; I therefore get a workman to prepare a lot of blocks, and keep them on hand for use as occasion requires.

Reader, if you try this plan once, I am persuaded that you will have no farther use for the papers and pasteboards you have been in the habit of using.

Lexington, Mo., January 3, 1849.

